# Synthetic cannabinoid-associated acute interstitial nephritis: An emerging cause of pediatric acute kidney injury? 

**DOI:** 10.5414/CNCS111063

**Published:** 2023-03-27

**Authors:** Ratna Acharya, Xu Zeng, Kiran Upadhyay

**Affiliations:** 1 Department of Pediatrics,; 2 Division of Anatomic Pathology, Department of Pathology, and; 3 Division of Pediatric Nephrology, Department of Pediatrics, University of Florida, Gainesville, FL, USA

**Keywords:** synthetic cannabinoid, interstitial nephritis, children, acute kidney injury

## Abstract

Synthetic cannabinoid (SCB) usage among children is a rapidly emerging public health concern in the United States. Acute kidney injury (AKI) is an uncommon manifestation of SCB usage, with acute tubular necrosis (ATN) as the predominant histology. Here we describe a 16-year-old adolescent who sustained severe non-oliguric AKI in association with SCB usage. Emesis, right flank pain, and hypertension were the presenting clinical features. There was no uveitis, skin rash, joint pains, or eosinophilia. Urinalysis showed absence of proteinuria or hematuria. Urine toxicology was negative. Renal sonogram showed bilateral echogenic kidneys. Renal biopsy demonstrated severe acute interstitial nephritis (AIN), mild tubulitis, and absence of ATN. AIN responded with pulse steroid followed by oral steroid. Renal replacement therapy was not required. Although the exact pathophysiology of SCB-associated AIN is not known, immune response elicited by the renal tubulointerstitial cells against the antigens present in the SCB is the most likely mechanism. A high index of suspicion for SCB-induced AKI is necessary in adolescents who present with AKI of unclear etiology.

## Introduction 

The usage of synthetic cannabinoids (SCB) among adolescents and young adults is a growing public health concern [1, 2, 3]. These designer drugs, such as “Spice” and “K2”, are full agonists of the cannabinoid receptors and may also act at serotonin and N-methyl-D-aspartate receptors [4]. They have higher affinity and are hence 2 – 800 times more potent than conventional Δ-9-tetrahydrocannabinol (THC), the main psychoactive constituent of cannabis [5, 6]. The common manifestations of SCB toxicity include tachycardia, agitation, vomiting, conjunctival redness, increased appetite, slurred speech, ataxia, and nystagmus [7]. Seizures, hyperthermia, hallucinations, delirium, and psychosis can also occur with severe overdose [7]. Renal manifestations are either unknown or not commonly reported. Acute kidney injury (AKI) secondary to usage of SCB is a relatively newly described entity and has been reported in adults [1, 8, 9, 10]. Srisung et al. [11] described 3 adults who sustained AKI in the setting of SCB usage, with 1 patient requiring hemodialysis. AKI due to SCB usage has also been recently described in adolescents; acute tubular necrosis (ATN) is the most common histology [1, 12]. Here, we describe a 16-year-old adolescent with severe AKI secondary to acute interstitial nephritis (AIN) without ATN, in association with SCB usage. To our knowledge, isolated AIN associated with SCB usage has not been described in the past. 

## Case report 

A 16-year-old Hispanic male presented to the emergency department with 5-day history of right flank pain and non-bloody, non-bilious emesis. There was no history of trauma. He reported loss of appetite but was able to drink. He was urinating well but had not had a bowel movement for 3 days. He denied dysuria, frequency, foamy urine, gross blood in urine, and urethral discharge. There was no history of fever, skin rash, joint pains, recent infections, autoimmune disease, systemic illness, or exposure to nephrotoxic agents. He particularly denied history of intake of non-steroid anti-inflammatory drugs (NSAIDs) and proton-pump inhibitors. 

Past medical history was not significant for major medical illnesses or prior history of AKI. Family history was negative for renal diseases. He was sexually active with one female partner. He suffered from depression but was not taking any anti-depressants. His school grades were failing, and he had been caught missing classes several times. He mentioned that he had been smoking cannabis and SCB for about a year with an influence from his peers, most of whom were over 18 years old. The last day of exposure was 2 days prior to presentation. 

Vital examination upon presentation showed temperature of 36.9 °C (98.4 °F), respiratory rate 18 per minute, pulse 110 beats per minute, and blood pressure 146/96 mmHg. His height was at 77^th^ percentile and weight at 40^th^ percentile. Capillary refill time was normal, and the mucous membranes were not dry. He exhibited right upper quadrant and right flank tenderness. There was no skin rash. The rest of the physical examination was normal. Initial blood urea nitrogen concentration (BUN) was 52 mg/dL, and serum creatinine was 9.2 mg/dL. There was no prior renal function test available for comparison. BUN peaked at 54 mg/dL and serum creatinine at 9.8 mg/dL on the 3^rd^ day of admission despite hydration. Other laboratory results are summarized in Table 1. Urinalysis showed specific gravity 1.006, pH 6, absence of protein, blood, glucose, and crystals, and negative nitrites and leukocytes. Urine eosinophils were not sent. Renal sonogram showed bilateral echogenic kidneys with right kidney measuring 12.5 cm and left kidney measuring 11.9 cm in length without hydronephrosis. Computed tomography of the abdomen and pelvis showed no acute abnormalities. 

**Figure 1:**
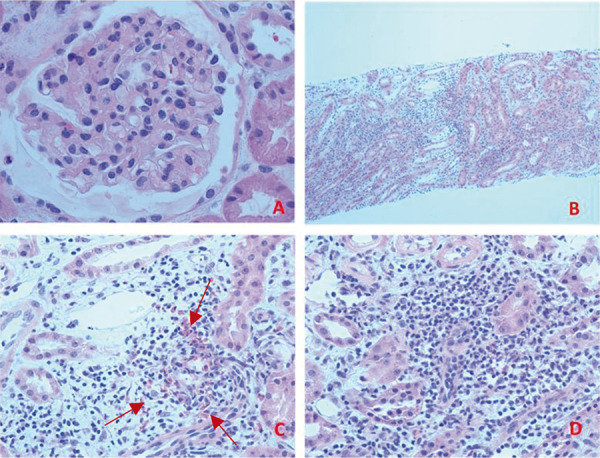
A: Glomerulus showing normal cellularity (H & E 40 × 10). B: Severe interstitial inflammation at cortico-medullary junction (H & E 10 × 10). C: Interstitial inflammation with many eosinophils (H & E 40 × 10). Arrows show many eosinophils identified by bilobed nuclei and characteristic red granular cytoplasmic stain. D: Interstitial inflammation and tubulitis (H & E 40 × 10).

A percutaneous renal biopsy was performed. Light microscopy showed 18 glomeruli with normal mesangial cellularity and matrix (Figure 1). There were no cellular crescents or areas of segmental sclerosis or necrosis. The tubular atrophy and interstitial fibrosis were very mild and patchy with ~ 5% overall tubular loss. There were foci of severe interstitial inflammation predominantly in the cortico-medullary junction, which was comprised of lymphocytes/mononuclear cells, many eosinophils (more than 10 eosinophils/high power field), and rare neutrophils. There was associated tubulitis. Tubules were otherwise intact. Vessels showed no active arteritis. Immunofluorescence showed diffuse, granular mesangial staining for trace IgA and C3 without specific glomerular staining for IgG, IgM, C1q, κ- or λ-light chains. Electron microscopy showed a normal trilaminar structure and thickness of the glomerular basement membrane. There were no immune deposits or tubulo-reticular inclusions. The majority of podocyte foot processes were intact. Proximal tubules showed intact brush borders and no evidence of ATN. 

Given biopsy findings of severe AIN, he was treated with a 3-day course of pulse intravenous steroid (1 g daily) with marked rapid improvement of renal function. Serum creatinine trended down to 1.9 mg/dL after 3 doses of pulse steroid. He required a few doses of isradipine for hypertension on an as-needed basis. Ophthalmologic examination obtained after completion of pulse steroid did not show uveitis. He did not require renal replacement therapy. He was then transitioned to oral prednisone 60 mg daily and was discharged home. A follow-up renal function test performed 2 weeks after discharge showed a serum creatinine of 0.9 mg/dL. Urine toxicology showed presence of cannabinoids when he returned for a follow-up. He admitted continued smoking of cannabis but reported abstinence from SCB since the hospital discharge. Extensive counseling on cessation of cannabis and SCB was done. Oral steroid was tapered off over 6 weeks. Renal function remained normal. Blood pressure returned to normal without antihypertensive agents. Urinalysis showed absence of proteinuria and hematuria. Upon follow-up at 3 months, he reported that he had discontinued using cannabis and SCB. A repeat ophthalmologic examination at that time showed no evidence of uveitis. 

## Discussion 

A systematic review of clinical effects of SCB reported acute anxiety and psychosis as the most common manifestations of SCB toxicity [13]. Other reported features are trouble thinking clearly, headache, dry mouth, vomiting, hypertension, tachycardia, hypothermia, irritability, ataxia, and seizures [7, 8, 14]. Chest pain, elevated troponin levels, electrocardiographic changes consistent with myocardial infarction have also been reported [15]. Renal manifestations are uncommon, and AKI has rarely been reported with SCB usage [1, 8, 10, 11, 16, 17], including in those without a prior history of renal disease [9]. In one multi-center, hospital-based registry of patients with isolated SCB intoxication, AKI was reported in 4% among 277 cases, and 6% had rhabdomyolysis [18]. Among those who sustain AKI, the most common extra-renal symptoms are intense nausea, persistent emesis, and abdominal pain [8, 17]. Indeed, cyclic vomiting, also known as cannabinoid hyperemesis syndrome, has been reported in patients consuming cannabis but without association with AKI [19, 20]. In 2012, Centers for Disease Control and Prevention reported 16 AKI cases among SCB users in six states in the USA [1]. XLR-11 was the synthetic cannabinoid identified from product samples and clinical specimens. The exact pathophysiology of SCB- or XLR-11-induced ATN is unknown. XLR-11 has been shown to primarily target the mitochondrial function in human proximal tubule (HK-2) cells (where endogenous expression of cannabinoid receptors occur) by causing transient hyperpolarization of the mitochondrial membrane and increased adenosine triphosphate (ATP)production, followed by Bax translocation from cytosol into mitochondria. This event is known to trigger energy-dependent apoptotic cell death pathways, as evidenced by increased caspase-3 activity and chromatin condensation [21]. Regarding AIN, one of the possible mechanisms includes antigen processing (such as XLR-11 and its metabolites) and presentation to local dendritic cells, which then activate T cells, and the subsequent effector phase of the immune response is mediated by various cytokines leading to hypersensitivity-type reaction in the renal tubulointerstitial cells. Other proposed mechanisms are the development of antibody to the exogenous antigen and then this complex eliciting the immune response, or haptenization of low molecular weight substance, such as XLR-11 and its metabolites, which then creates an immune response [22]. It is also possible that these SCBs may be adulterated with other known nephrotoxins or previously unknown newer nephrotoxins that could elicit immunological response by the tubulointerstitial cells, leading to AIN [23]. Most of the time, it is difficult to know the exact constituents of these SCBs, given that they are illegally manufactured. Since we were not able to analyze the ingredients of the SCB taken by our patient, the exact trigger for AIN remains uncertain. However, since there was no concurrent usage of other known nephrotoxic agents, such as NSAIDs, it seems reasonable to assume a potential causal association of AIN with SCB. This is unlike other reports that described ATN as the predominant renal histology [8]. 

Among high school students aged 17 – 18 in the United States, 2.9% reported current SCB use, and 1.4% reported using SCBs on ≥ 3 days in the past month. They were also likely to use polysubstance such as cannabis, lysergic acid diethylamide, cocaine, heroin, and non-medical opioids [24, 25]. In a prospective, observational study of adolescents with SCB intoxication, coma and seizures were more likely than in adolescents with cannabis intoxication [7]. Data on the incidence of SCB-associated AKI in adolescents is scarce. Buser et al. [12] described two adolescents, 15 and 17 years old, who sustained AKI secondary to SCB usage. Both had flank pain and emesis. The renal biopsies showed features of predominant ATN with mild interstitial nephritis and peritubular capillaritis [12]. Both were treated with steroids; one required hemodialysis for oliguria. Similarly, another study [1] reported that out of 8 patients (in a total cohort of 16 patients with SCB-associated AKI) who underwent renal biopsy, 6 had ATN and 3 had AIN; it is not very clear from their report whether these patients had isolated ATN or AIN or combined ATN and AIN. Our report is unique given that the renal biopsy showed isolated severe AIN without ATN, but this could also be due to a sampling error. Clinically, there was no oligoanuria, and since the AKI improved rapidly with pulse steroid, dialysis was not required. 

As described in this report, concurrent usage of cannabis and SCB is commonly seen. Gunderson et al. [14] conducted a survey in 2014 of 42 young-adult current cannabis users about their SCB usage and found that 90% were familiar with the SCB products, half had smoked SCB in the past, and a quarter of them reported current use of SCB. Most common reasons for using SCB was to avoid drug detection in urine tests and to achieve a new “high”. Urine drug screening does not detect SCBs as the SCBs and their metabolites do not cross-react with Δ-9 THC [26]. Arnston et al. [26] described two validated ELISAs designed to detect the two common SCBs in urine, and this may aid in the early diagnosis of SCB consumption. XLR-11 N-pentanoic acid metabolite can also be measured in toxicological laboratory work-up in both urine and blood via gas chromatography/mass spectrometry or liquid chromatography/time-of-flight mass spectrometry [1]. Whether severe AKI is a manifestation of combined cannabis-SCB usage or SCB only needs to be investigated in future studies. Cannabis alone has not been shown to increase the risk of AKI in patients with advanced chronic kidney disease (CKD) [27]. 

Despite normalization of renal function in most reported patients with SCB-associated AKI, repeat insults of AKI with ongoing SCB usage could be a risk factor for development of CKD. Indeed, patients with a history of repeated any-cause AKI episodes have a higher risk of developing CKD and end-stage renal disease (ESRD) [28]. In the subset of patients who continue to consume SCBs, the risk of CKD and ESRD is unknown and would be an interesting area of study for future investigators. 

This report highlights the importance of a high index of suspicion of SCB usage when encountering an adolescent with AKI of unclear etiology, especially in association with emesis and hypertension. Timely evaluation with renal biopsy and initiation of steroid therapy may expedite the renal recovery and prevent irreversible damage. 

## Funding 

The authors received no funding for the purpose of this study. 

## Conflict of interest 

The authors declare no conflict of interest. 

**Table 1.** Blood and urine investigations.

Sodium (meq/L)	136
Potassium (meq/L)	4.3
Bicarbonate (meq/L)	21
Calcium (mg/dL)	9.2
Phosphorus (mg/d)	5.8
White blood cell (10^9^/L)	13.2
Hemoglobin (g/dL)	14.1
Platelet count (10^9^/L)	332
Neutrophils, lymphocytes, eosinophils	78.4%, 9.9%, 0.4%
Liver function test	Normal
Lactate dehydrogenase (IU/L)	131 (135 – 225)
Lipase (U/L)	9 (0 – 70)
Creatine kinase (U/L)	48 (22 – 198)
Iron saturation	44% (> 20%)
Intact parathyroid hormone (pg/mL)	57 (12 – 88)
Complement 3 (mg/dL)	113 (87 – 200)
Complement 4 (mg/dL)	28 (13 – 50)
Antinuclear antibody	Negative
Antinuclear cytoplasmic antibody	Negative
Anti-glomerular basement membrane antibody	Negative
Human immunodeficiency virus	Negative
Hepatitis panel	Negative
Syphilis screen	Negative
Urine chlamydia and gonorrhea	Negative
Urine myoglobin	Negative
Urine culture	Negative
Urine protein creatinine ratio	0.18 mg/mg
